# Localized asymptomatic cerebellar abscess after penetrating brain injury by wooden foreign object with adequate antibiotics administration: A case report

**DOI:** 10.1016/j.ijscr.2020.05.058

**Published:** 2020-05-30

**Authors:** Quri Meihaerani Savitri, Corinne Prawira Putri, Kevin Jonathan Gunawan, Dini Lukita Hapsari, Iwan Sidharta, Pandu Wicaksono

**Affiliations:** a Emergency Department, Semen Gresik Hospital, Gresik, Indonesia; b Department of Surgery, Semen Gresik Hospital, Gresik, Indonesia

**Keywords:** Cerebellar abscess, Occupational accident, Penetrating brain injury, Wooden foreign body

## Abstract

**Introduction:**

Brain abscess is a compilation of pus enclosed in capsule as a result of focal infection in brain parenchyma. It is one of several complications found in patients who suffered penetrating brain injury.

**Case presentation:**

Thirty-four-year-old man suffered a penetrating brain injury after a 50 cm piece of wood penetrated through his facial skull and ended its tip in his cerebellum, the wood priorly ejected from a moulding machine. As a consequence, he had to undergo a craniotomy procedure to remove the foreign body object and its debris. Following the surgery, adequate antibiotics were administered. Postoperative enhanced head CT revealed a cystic mass formation in the left hemisphere of cerebellum, measured 20 × 28 mm with blood density lesions and a visible ring enhancement. These features suggested a cerebellar abscess. The follow-up enhanced head CT later demonstrated that the size, shape, and location of the abscess were relatively consistent with the previous head CT.

**Discussion:**

Penetrating brain injury (PBI) is the most life-threatening head trauma. Although the prevalence number was low compared to other head traumas, its morbidity and mortality number were higher. Brain abscess formation is one of the many PBI complications. Due to direct inoculation of foreign body and its debris, PBI commonly leads an infection process. However, the infection process is supposed to be overcome by administering broad-spectrum antibiotics prophylactically. This case presented an inevitable brain abscess despite of the adequate antibiotics administration.

**Conclusion:**

Despite adequate antibiotics has been administered, cerebellar abscess after penetrating brain injury is still found challenging to manage. Therefore, holistic-multidisciplinary approaches are needed.

## Highlights

• Penetrating Brain Injury (PBI) incidence rate is lower than other type of head traumas, but it's the most hazardous one.
• Non enchanced head CT (NECT) is beneficially helpful for the surgeons to construct surgical plan and to estimate the prognosis related to patient's condition.
• Brain abscess is commonly found as PBI complication, it can appear 2–4 weeks, in some cases may delayed to 2–3 months after the time of injury.
• The broad-spectrum prophylactic antibiotics is important to control the infection, though abscess formation may be inevitable.

## 1 Introduction

Penetrating brain injury (PBI) comprises all head traumas caused by objects which penetrate the skull into the brain structures and may cause extensive damage to the structures inside [1]. In western nations, this type of injury is closely associated to projectile (gunshot) accidents. Meanwhile in most of developing countries, this type of injury is related to non-projectile (sharp object) accidents which usually found in victims of occupational accidents or traffic accidents [2]. Gresik known as an industrial city in East Java. Therefore, occupational-related-accident cases were more commonly found in our Emergency Room (ER) [3]. Most of them happened due to lack of awareness of wearing personal protective equipments. However, PBI case in our ER was pretty rare, since most of the patient could not reach the ER door alive due to the extensive ongoing bleeding. Those who survived may suffer long term neurological defects [4].

Brain abscess is a complication that closely related to PBI. It is a result from infection process where pus were collected then were enclosed in well-vascularized capsule [5]. The source of infection itself was from direct inoculation of foreign body, either embeded or passed through the brain structures [5]. After all, direct inoculation is mostly came from either the traumatic brain injury itself or the neurosurgical procedures [5]. However, the frequency of direct-inoculation-caused brain abscess is rare, only 8–19% from all brain abscess cases [5]. We present a rare case of localized asymptomatic cerebellar abscess following accidental penetration of wooden foreign object which needs continous monitoring and adequate managements from multidisciplinary team.

This work has been reported in line with the SCARE criteria [6].

## 2 Case report

### 2.1 Initial presentation

**Fig. 1:**
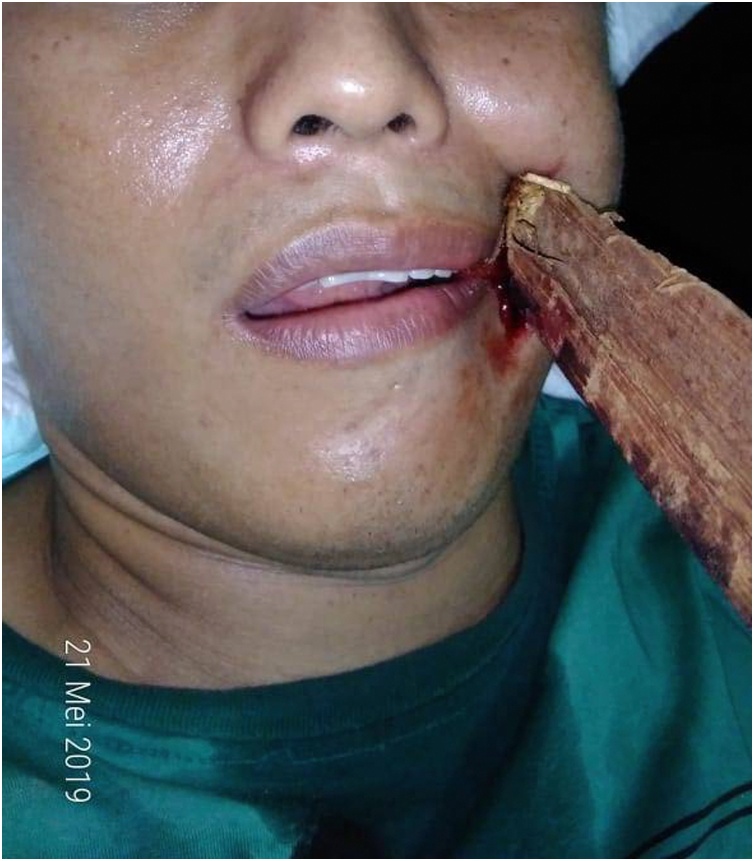
Initial presentation of patient in ER. The wooden foreign body penetrated through the left nasolabial fold.

A 34-year-old man suffered a stab wound by lumber ejected from the machine during the moulding process, then penetrated through his left nasolabial fold. At that moment, the patient wore safety eyeglasses without a full-face shield. Then the lumber was shortened by his colleague and the remaining 30 cm lumber was left embedded on the man's face. The man was brought into our ER by ambulance and was administered as trauma patient. The patient complained about the pain in his left cheek, moderate bleeding was identified from the site of penetration. Loss of consciousness, impaired vision, nausea, and vomiting were denied. On his arrival at the ER, the patient was alert and cooperative (Glasgow Coma Scale (GCS) score 15). The patient has no history of allergies or past illness. Patient's vital signs were within normal limits. On neurological examination, a decreased blink reflex on his left eye was found, but signs of basilar skull fracture (e.g. raccoon eyes, rhinorrhea, and otorrhea) and muscle weakness or sensory impairment in all 4 limbs were not found (Medical Research Council (MRC) Scale 5/5) (Fig. 1).

### 2.2 Initial imaging

**Fig. 2:**
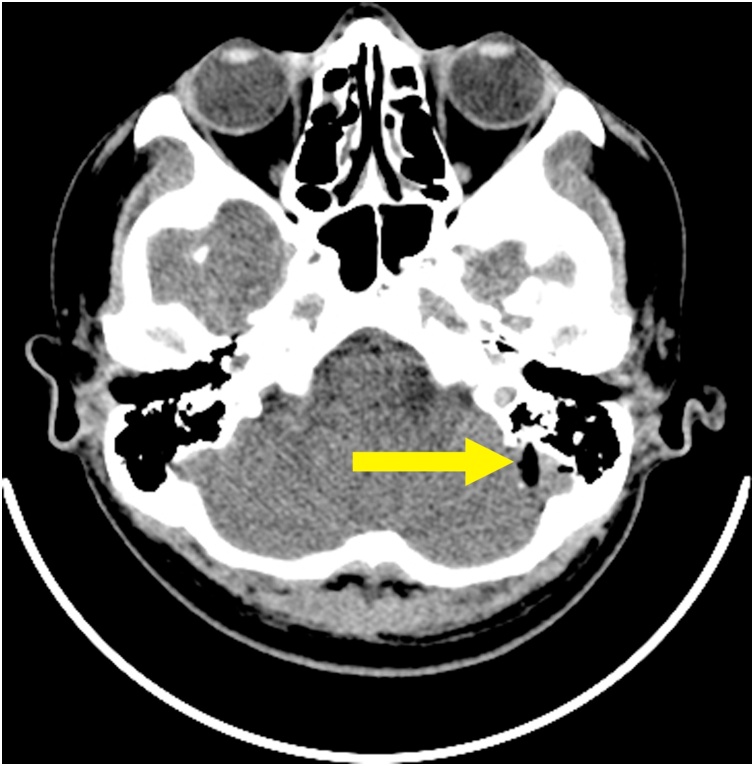
Preoperative NECT described a low-attenuated area in the left lobe of cerebellum (yellow arrow).

**Fig. 3:**
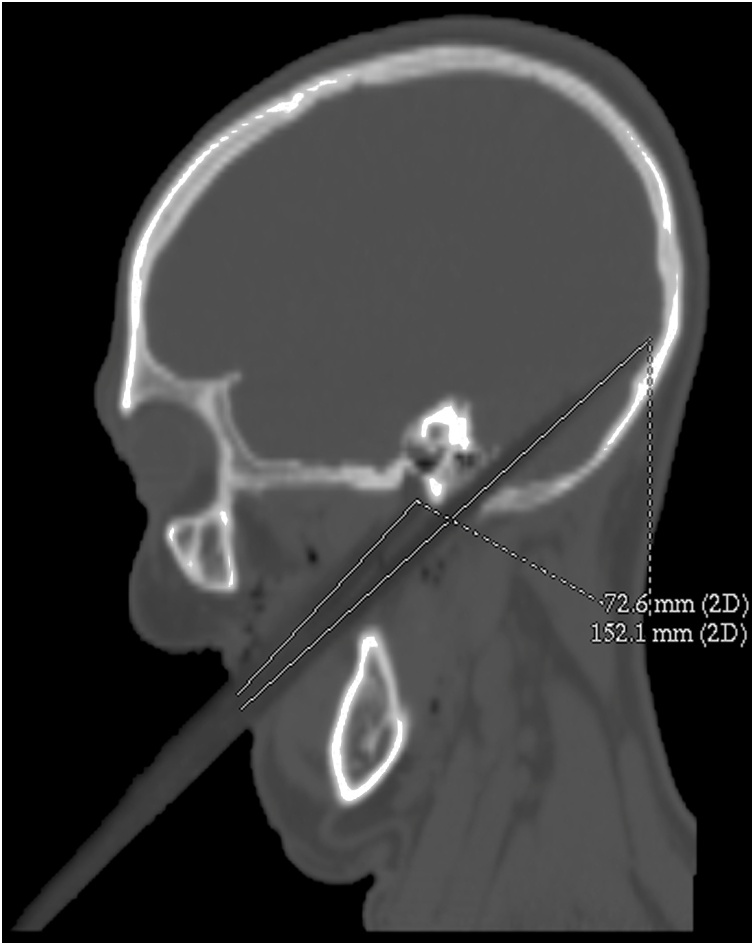
Preoperative NECT described a low-attenuated area, suggesting the location of foreign wooden body, which are embedded *in situ*, and the length of foreign wooden body was 152.1 mm.

**Fig. 4:**
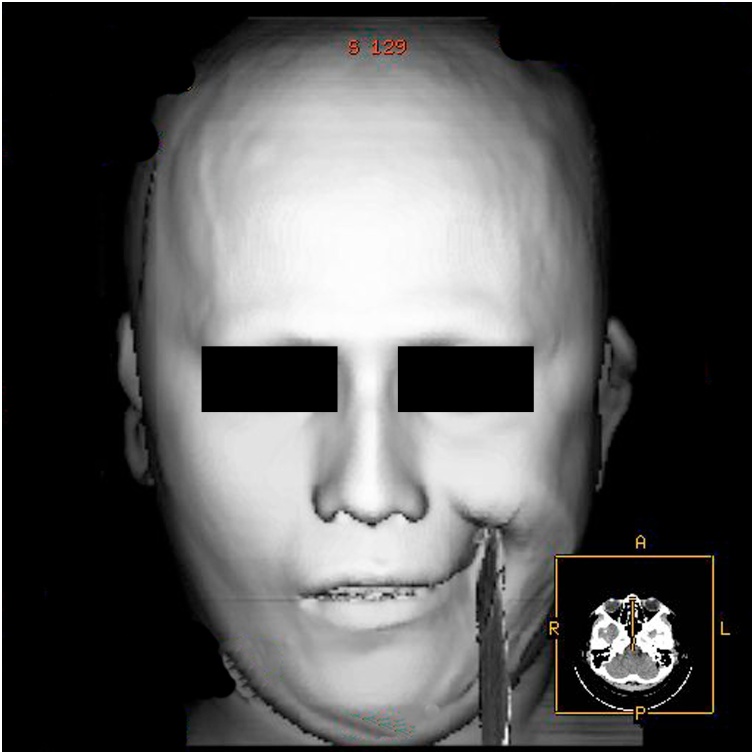
Preoperative 3D head CT reconstruction showed foreign the foreign wooden body penetrated the man's left nasolabial fold.

Non-enchanced head CT (NECT) showed a low-attenuation area. It described a wooden foreign object penetrated the cerebellum. The length of the object was 152.1 mm embedded *in situ* (Fig. 2, Fig. 3, Fig. 4).

### 2.3 Surgical process

**Fig. 5:**
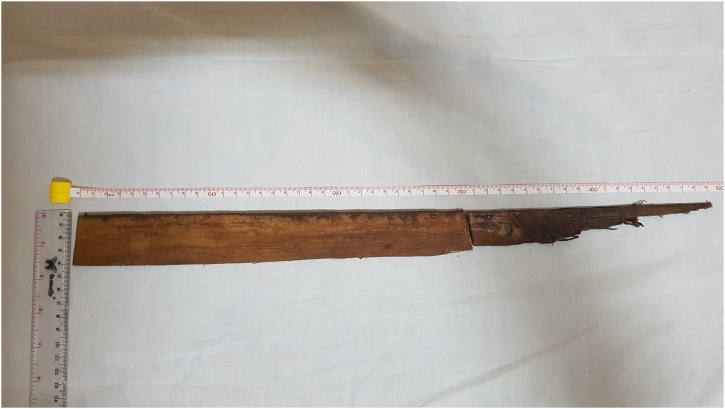
The total length of the foreign wooden body was 49.4 × 4 cm, divided into two parts which are *in-situ* part (19 × 2.6 cm) and *ex-situ* part (30.7 × 4 cm).

The surgical process was conducted by a collaboration of head and neck surgeon, neurosurgeon, and anesthesiologist. Prior to the surgery, 2 grams of ceftriaxone were administered as prophylactic antibiotics. The patient was positioned in a park-bench position. A C-shaped incision was made over the retrosigmoid area. After the muscles were uncovered, initial burr hole was made and then it was enlarged using bone rongeur forceps superiorly and laterally until it reached the sinus border. Dura mater was incised linearly and the bulging brain was identified. Corticotomy was performed to reduce brain’s buldging and to minimize difficulties so the neurosurgeon could evaluate the condition of the facial nerve and vestibulocochlear nerve. Bone defects were palpated on the petrous bone at the cerebellopontin angle. The extraction of the wooden foreign object was performed from the site of entrance wound, then followed by debridement process and exploration of its surrounding area. Once the object was clearly removed, bleeding was controlled using electrocautery. After repeated irrigation with normal saline, the bone defect was closed with the muscle patches and glued by Beriplast®. Drains were placed on the cisterna and on the entrance wound in the left nasolabial fold. Watertight dural closure was performed, then followed by adequate scalp closure. The total length of the foreign wooden body is 49.4 × 4 cm, the measurement of *in-situ* part was 19 × 2.6 cm, while the measurement of *ex-situ* part was 30.7 × 4 cm (Fig. 5).

### 2.4 Postoperative course

**Fig. 6:**
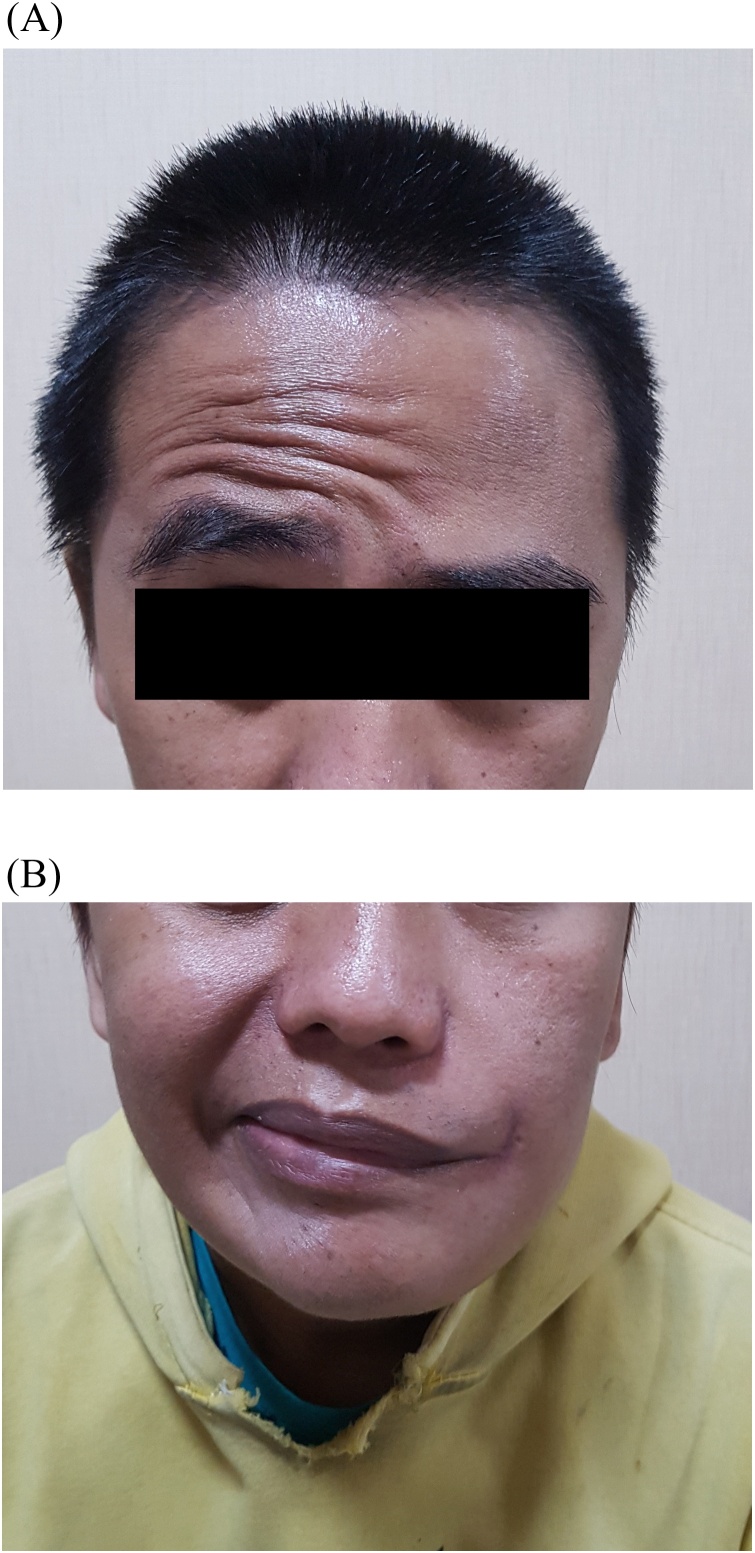
A & B. Postoperative neurological examination, facial nerve palsy were found.

Patient was observed in the ICU for 24 hours. Ceftriaxone (1 g q12 h) was administered for 6 days after the surgery completed. Patient was not showing any clinical signs of infections (e.g. fever, persistent headache, altered state of consciousness, seizure, nausea and vomiting, and motor or sensory impairments). Patient’s laboratory results were showing a slight increased WBC (18,060 normal parameter: 4,000–11,000). The patient postoperative course was uneventful and he was discharged after 6 days with GCS 15 and clean-healthy postoperative wound. On his neurological examination, patient complained a slight tingling sensation on his left face, lagophthalmos on his left eyelid, and mild difficulty to chew by his left side of mouth. Facial nerve palsy and paresthesia of the trigeminal nerve were found (Fig. 6A & B). Co-amoxiclav 500 mg three times a day orally was given after patient discharge from hospital for 5 days. Followed up 10 days after the surgery, patient did not show any symptoms of infection. However, facial nerve palsy and paresthesia of the trigeminal nerve remained consistent. The patient was referred to a physiatrist for physiotherapy and received short wave diathermy (ENRAF NONIUS Curapuls 970 in continuous mode, frequency generator 27.12 MHz, pulse repetition frequency 50 Hz, for 10 minutes) and Galvanization (ENRAF NONIUS Curapuls 591 in continuous mode, frequency 50 Hz). The therapy was carried out for 6 sessions in 12 days.

**Fig. 7:**
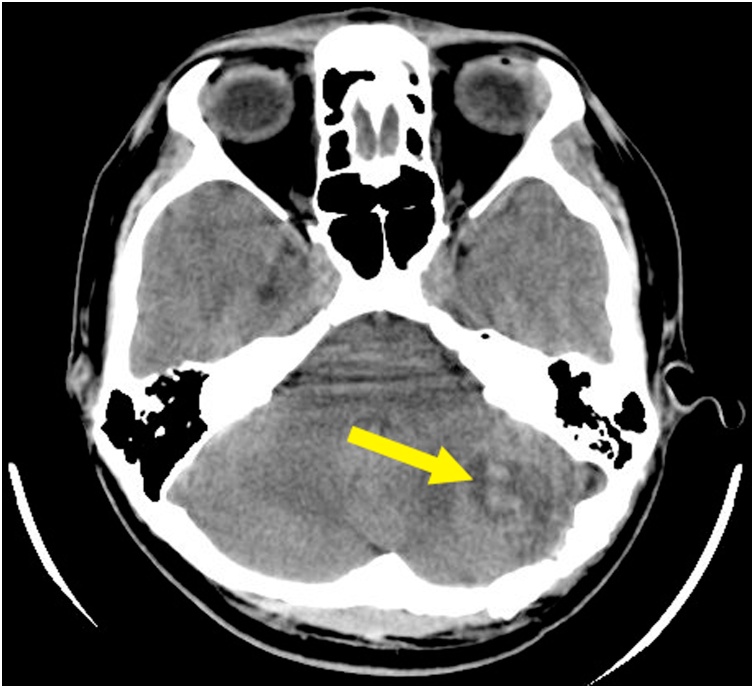
Thirty day postoperative enhanced head CT images showed a formation cystic mass in the left lobe of the cerebellum with blood density lesions and visible ring enhancement. These features suggested a formation of cerebellar abscess.

**Fig. 8:**
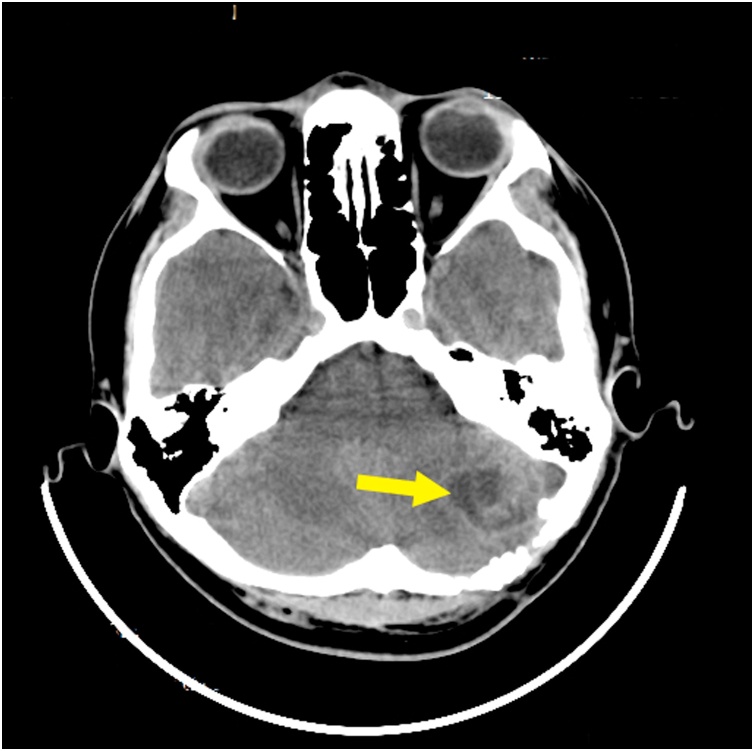
Three month postoperative enhanced head CT images showed the brain abscess was relatively consistent with its previous size, shape, and location.

The tingling sensation on patient’s left face was completely reduced and minimal improvement of facial nerve motor function was noted. Thirty days after the surgery, an enhanced head CT was performed and a cystic mass in the left cerebellum lobe was found. It measured 20 × 28 mm with blood density lesions and visible ring enhancement (Fig. 7). These features suggested a brain abscess, particularly in the left lobe of the cerebellum. Responding to these features, third generation of cephalosporin (Cefixime 200 miligrams q12 h) and Metronidazole (500 miligrams q8h) were given to the patient for six weeks. Followed up three months after the surgery, patient didn’t complain any symptoms, including cerebellar abscess symptoms, such as nystagmus, ataxia, vomiting, and dysmetria. His laboratory finding was within normal limits and signs of infection were not found (White Blood Cell Counts 6,700 normal parameter : 4,000–11,000, Erythrocyte Sedimentation Rate 5 normal parameter : 0–15, Quantitative C-Reactive Protein 0.25 normal parameter : < 10). Later on, enhanced head CT was repeated and revealed that abscess’ size, shape, and location were consistent compared to previous enhanced head CT’s result (Fig. 8).

## 3 Discussion

Penetrating brain injury (PBI) is the most life-threatening head trauma [4]. The prevalence of the event represents only around 0.4–1.5% of all head trauma events, only 10% of patients were able to survived until their arrival at the hospital [2,4]. Some patient immediately died in the ER (The mortality ranges between 23–93%), the other suffered long-term neurological complications (The complications rate as high as 87–100%) [4]. While in western country, PBI was mostly caused by projectile injury such as gunshot, while in most developed and third world countries, PBI was more commonly caused by non-projectile injury. Depending on the variety of shape and sharpness of the weapons or objects from the accidents, they may penetrate the skull, dura mater, and reach into brain structures [4]. Penetrating wooden foreign body is one of non-projectile injury which is extremely uncommon, most cases were found in victims of traffic accidents or occupational accidents.

Speed ​​is the primary determinant of kinetic energy through the equation EK = ½ mv^2^. Projectile injury has an higher impact with velocity over 100 m/second, while non-projectile injury has lower impact with velocity less than 100 m/second [7]. Trauma due to wooden foreign body belongs into low-velocity impact and provokes localized tissue damages along the object path [8]. The damage severity of low-velocity object is less severe due to its lesser kinetic energy. Moreover, the low-velocity impact has better prognosis relatively [9].

The role of imaging is crucial to determine the initial assessment of injury's type and severity, to set up the surgical plan, the type of surgery, including the size and the location of craniotomy, the foreign body's extraction routes, the decision of non-operative managements, as well as to estimate the early and advanced complications and the overall prognosis [9]. The findings on radiological examination are critical, including the position of wound's entry and exit, the intracranial wound's fragments, the wound's pathways, and the condition of adjacent blood vessels and other structures, the acquired traumas from the injury, such as trans-ventricular trauma, basal ganglia trauma, intraparenchymal brain trauma, multi-lobe trauma, basal cistern effacement, brain herniation, and other associated mass effect traumas [10]. Plain skull x-ray or CT are indicated in patients with head trauma, however, head CT is much reliable to diagnose PBI [1]. Non-enhanced head CT (NECT) is a preferred imaging modality because it consumes less time, able to identify fractures, accurately locates any intracranial foreign bodies and fragments, evaluates the extent of brain damage, notices any involvement of major blood vessels or other intracranial structures, detects intracranial hematomas and mass effects, and provides 3D reconstruction imaging [6,9,11,12]. 3D reconstruction is helpful to comprehend the accurate size, length, direction, and position of the foreign body in various views. These features are beneficial in conceiving the surgical procedures [6].

The Canadian CT Head Rules (CCHR) and National Institute for Clinical Excellence (NICE) recommend the clinical criteria to distinguish patients who needs immediate head CT (within 1 hour) or delayed head CT within a reasonable period. High risk factors, including GCS < 13 at initial examination or suspicion of a depressed skull fracture, require immediate head CT within an hour since the patient's arrival at the ER. When a patient presents with one or more moderate risk factors, including retrograde amnesia > 30 minutes, dangerous trauma mechanism, or age > 65 years, the head CT can be delayed within 8 hours since the admission time [1].

In CT, plant-origin foreign body usually appears as non-attenuated to low-attenuated area due to high air content, while metal-based foreign body exhibits high-attenuated area [13]. However, wooden foreign body's appearance depends on their water content. Fresh-cut wood usually has higher water content and may mimics the appearance of its surround soft tissues and muscles. On the contrary, processed wood commonly has lesser water content and may mimics the appearances of its surround air or fat [14].

In all forms of trauma, the Advanced Trauma Life Support (ATLS) guidelines are essential for patient management. The focus of initial resuscitation is to prevent hypoxia and hypotension by maintaining adequate airway, breathing, and circulation. During resuscitation, foreign body should never be forcibly removed outside the operating theater. Blind removal can cause severe hemorrhage from major blood vessels, leading to subdural hematoma and intracerebral hemorrhage as complications [2]. The distal portion of the foreign body should be shortened cautiously when it causes a hindrance during transport process or imaging procedures, particularly entering CT Scanner process [6]. In addition, the remaining foreign body gives tamponade effect to control the bleeding. In this case, maintaining the wooden foreign body on the patient's face was the correct action. Foreign body removal is ideally proceeded in the operating theater under general anesthesia.

This case involves multidisciplinary teamworks, started from emergency team in initial management, radiologist in diagnostic imaging procedure, clinical pathologist in diagnostic laboratory procedure, anesthesiologist, head and neck surgeon, and neurosurgeon in surgical procedures. The goals of surgical procedures in this patient were to remove the foreign body, necrotic tissues, debris, and other potential contaminants, to evacuate any hematomas and to control the bleeding, as well as to prevent cerebrospinal fluid leakage [4]. Extraction of foreign body is performed without making exaggerated swinging motions to prevent further damage [4]. Delayed surgery, more than 12 hours since the time of injury, increases the risk of infection [4]. Patient with fixed dilated pupils and poor CT findings are generally not recommended for surgery because of the poor prognosis. Poor head CT findings include brain stem injury, bilateral hemispheric injury, multilobar or transventricular injury, subarachnoid hemorrhage, extensive intracerebral hemorrhage, midline shift, and the presence of "tram-track sign" (bleeding on both sides of the trajectory of perforating injury) [15]. Surgical procedure is recommended to patient with GCS 6 or greater, while unequal or reacting pupils and space occupying hematoma, patient with GCS below 6, surgical option is still controversial [14].

Complications of skull penetration trauma are classified into early stages (< 1 week) and advanced stages (> 1 week) [4]. Brain abscess preceded by infection process belongs to advanced complications. Brain abscess formation has three causes, which are extensive infection from adjacent pericranial site (sinuses, middle ear, or dental infection), hematogeneous spread from distant focus of infection (lung abscess, bacterial endocarditis, intraabdominal and pelvic infection, and skin infection), and direct inoculation following head trauma or neurosurgery [5]. In PBI case, brain abscess is caused by direct inoculation of foreign body and its debris, such as the projectile particles, hair, skin, or bone fragments entering the intracranial cavity [4]. The formation of brain abscess habitually occur around the brain defect part or debris and noticed within 2–4 weeks after initial injury [4]. Meanwhile delayed intracranial abscess formation might happens within 2–3 months [16]. Patient with brain abscess may suffers increased high intracranial pressure symtoms (e.g. headache, vomiting, and altered mental status), focal neurologic deficits, and fever [5]. The severity of the symptoms depends on the origin of infection, site, size, number of lesions, specific brain structures involved, adjacent structure disturbance, and any secondary cerebral injury [5]. Enhanced head CT is essential to diagnose brain abscess [17]. The typical finding on enhanced head CT is hypodense lesion with contrast-enhanced ring [4].

*Staphylococcus aureus*, *Staphylococcus epidermidis,* and gram-negative bacteria are the most common pathogens [4]. Administration of broad-spectrum prophylactic antibiotics is neccessary while the clinician is waiting the culture and antibiotic sensitivity reports from the pathogens of pus [4].

In this case, patient’s brain abscess conditions fulfill the criteria to go under medical treatment. Those criteria are small size abscess (< 2.5 cm), patient's good initial clinical condition (GCS > 12), and the etiology of the abscess is confirmed. The antibiotics’ choices are based on their ability to pass through the blood-brain barrier and the blood-CSF (Cerebrospinal fluid) barrier to achieve adequate concentration [5,15]. Initial therapy should be started with empirical-broad-spectrum antibiotics which have the ability to cross the blood-brain and blood-CSF barriers in adequate concentrations. The adjuncted antibiotics should be added to cover anaerobic pathogens [5]. Penicilin, ampicilin, cefuroxime, chloramphenicol, co-trimoxazole, ceftazidime, and metronidazole have been known to achieve therapeutic concentration [5]. Nowadays, third-generation cephalosporin, metronidazole, and vancomycin are widely use [5]. However, the cause of brain abscess formation in this patient is still unknown, despite of given adequate antibiotics.

## 4 Conclusion

Despite of adequate antibiotics administration, cerebellar abscess after penetrating brain injury is challenging to manage. Holistic multidisciplinary approaches, including eligible clinicians, pharmacological knowledge, and surgical skills, contribute in patient’s better outcomes and prognosis.

## Funding

No funding or grant support.

## Ethical approval

This case report has been approved by Semen Gresik Hospital Ethical Committee.

## Consent

Patient gave consent to share the information about himself (photograph and article) to appear on a journal article. The information will be published without name attach and every attempt will be made to ensure anonymity. The information may be published in a health journal which is read worldwide.

## Registration of research studies

1. Name of the registry:-
2. Unique identifying number or registration ID:-
3. Hyperlink to your specific registration (must be publicly accessible and will be checked):-

## Guarantor

Quri Meihaerani Savitri, MD.

## Provenance and peer review

Not commissioned, externally peer-reviewed.

## CRediT authorship contribution statement

**Quri Meihaerani Savitri:** Writing - original draft, Project administration, Visualization. **Corinne Prawira Putri:** Conceptualization, Investigation, Writing - original draft. **Kevin Jonathan Gunawan:** Writing - review & editing. **Dini Lukita Hapsari:** Writing - review & editing. **Iwan Sidharta:** Supervision. **Pandu Wicaksono:** Supervision.

## Declaration of Competing Interest

The following authors have no conflict of interest: QMS, CPP, KJG, DLH, IS, PW.

## References

[1] Temple N., Donald C., Skora A., Reed W. (2015). Neuroimaging in adult penetrating brain injury: a guide for radiographers. J. Med. Radiat. Sci..

[2] Lan Z.G., Richard S.A., Li J., Yang C. (2018). Nonprojectile penetrating iron rod from the oral cavity to the posterior cranial fossa: a case report and review of literature. Int. Med. Case Rep. J..

[3] Pusat Badan, Provinsi Statistik, Timur Jawa (2015). Number of Establishment and Labour Force by Regency/City in East Java, 2015. https://jatim.bps.go.id/statictable/2018/02/08/886/jumlah-perusahaan-dan-tenaga-kerja-menurut-kabupaten-kota-di-jawa-timur-2015.html.

[4] Vakil M.T., Singh A.K. (2017). A review of penetrating brain trauma: epidemiology, pathophysiology, imaging assessment, complications, and treatment. Emerg. Radiol..

[5] Miranda H.A., Castellar-Leones S.M., Elzain M.A., Moscote-Salazar L.R. (2013). Brain abscess: current management. J. Neurosci. Rural Pract..

[6] Agha R.A., Borrelli M.R., Farwana R., Koshy K., Fowler A., Orgill D.P., For the SCARE Group (2018). The SCARE 2018 statement: updating consensus surgical CAse REport (SCARE) guidelines. Int. J. Surg..

[7] Li X.S., Yan J., Liu C., Luo Y., Liao X.S., Yu L. (2017). Nonmissile penetrating head injuries: surgical management and review of the literature. World Neurosurg..

[8] Young L., Rule G.T., Bocchieri R.T., Wlilko T.J., Burns J.M., Ling G. (2015). When physics meets biology: low and high-velocity penetration, blunt impact, and blast injuries to the brain. Front. Neurol..

[9] Sim S.K., Theophilus S.C., Noor Azman A.R. (2014). Multiple nail gun penetrating head injury: a case report. IMJM.

[10] Kazim S.F., Shamim M.S., Tahir M.Z., Enam S.A., Waheed S. (2011). Management of penetrating brain injury. J. Emerg. Trauma Shock.

[11] Agrawal A., Reddy V.U., Kumar S.S., Hegde K.V., Rao G.M. (2016). Transorbital orbitocranial penetrating injury with an iron rod. Craniomaxillofacial Trauma Reconstr. Open.

[12] Jeon Y.H., Kim D.M., Kim S.H., Kim S.H.W. (2014). Serious penetrating craniocerebral injury caused by a nail gun. J. Korean Neurosurg. Soc..

[13] Wakisaka H., Takahashi H., Ugumori T., Motoyoshi K., Takagi D. (2010). A case of a wooden foreign body penetrating the oral cavity and reaching the posterior neck. Inj. Extra.

[14] Kim D.H., Park E.S., Seong H.Y. (2016). A case of intracranial wooden foreign body: mimicking pneumocephalus. Korean J. Neurotrauma.

[15] Rosenfeld J.V., Bell R.S., Armonda R. (2014). Current concepts in penetrating and blast injury to the central nervous system. World J. Surg..

[16] Milton J., Awuor V. (2017). A unique presentation of an intracranial abscess secondary to retained projectile after debridement with dural closure. Cureus.

[17] Muzumdar D., Jhawar S., Goel A. (2011). Brain abscess: an overview. Int. J. Surg..

